# Physiological Phenotypes in Comatose ICU Patients: A Retrospective Multidimensional Analysis

**DOI:** 10.3390/diagnostics16172876

**Published:** 2026-09-07

**Authors:** Pompiliu Mircea Bogdan, Alina Pleșea-Condratovici, Roxana Elena Bogdan-Goroftei, Letiția Doina Duceac, Adina Oana Armencia, Marius Gabriel Dabija, Camer Salim, Vlad-Andrei Dabija, Pantelie Nicolcescu, Carmen Laura Cristescu-Budală, Eloise Raisa Barbu, Alina Mihaela Calin

**Affiliations:** 1 Doctoral School of Biomedical Sciences, Faculty of Medicine and Pharmacy, Research Center in the Medical-Pharmaceutical Field, “Dunarea de Jos” University of Galati, 800008 Galati, Romania; pompiliu.bogdan@ugal.ro (P.M.B.); raisauibariu@gmail.com (E.R.B.); 2 Faculty of Medicine and Pharmacy, Research Center in the Medical-Pharmaceutical Field, “Dunarea de Jos” University of Galati, 47 Domneasca Street, 800008 Galati, Romania; alina.plesea@ugal.ro (A.P.-C.); nicolcescu@yahoo.com (P.N.); laura.cristescubudala@gmail.com (C.L.C.-B.); alina.calin@ugal.ro (A.M.C.); 3 “Grigore T. Popa” University of Medicine and Pharmacy Iasi, 16 Universitatii Street, 700115 Iasi, Romania; adina.armencia@umfiasi.ro (A.O.A.); mariusdabija.md@gmail.com (M.G.D.); 4 Faculty of Medicine, “Ovidius” University of Constanta, 900470 Constanta, Romania; salimcamer@yahoo.com; 5 “Saint Spiridon” County Emergency Hospital, 1 Independenței Boulevard, 700111 Iasi, Romania; vladdabija10@gmail.com

**Keywords:** coma, intensive care units, biomarkers, inflammation, blood coagulation, cluster analysis, mortality

## Abstract

**Background/Objectives**: Comatose patients admitted to intensive care represent a heterogeneous critical population, in which prognosis can be influenced by the interaction between inflammation, coagulation, tissue damage and respiratory dysfunction. This study aimed to identify physiological phenotypes in comatose ICU patients and evaluate their association with ICU outcome. **Methods**: A retrospective observational study was performed on 227 adult comatose patients admitted to the ICU. Clinical variables, inflammatory and coagulation markers, LDH, blood gas parameters, APACHE and SOFA scores and ICU outcome were analyzed. Spearman correlations, hierarchical cluster analysis, Kruskal–Wallis tests, Dunn post hoc, effect sizes and adjusted logistic regression were used. **Results**: ICU mortality was 65.6%. Cluster analysis suggested three tentative phenotypes: inflammatory–coagulopathic, relatively stable, and a severe respiratory–inflammatory profile. Differences between clusters were mainly determined by D-dimers, CRP, procalcitonin, INR, and LDH, while APACHE and SOFA scores did not differ significantly between phenotypes. ICU outcome differed significantly between clusters (χ^2^ = 18.370; *p* < 0.001; Cramer’s V = 0.284), with the maximum mortality in Cluster 3. After adjustment, cluster membership did not remain an independent predictor of mortality. **Conclusions**: Comatose patients admitted to the ICU can be classified into exploratory physiological phenotypes, associated with different clinical outcomes in the unadjusted analysis. These results support the utility of multidimensional biological assessment but require external validation before clinical application.

## 1. Introduction

Patients admitted to intensive care units represent a highly heterogeneous population, in which prognosis is influenced by the interaction between organ dysfunction, systemic inflammation, coagulation abnormalities, respiratory impairment, and metabolic alterations [1]. In clinical practice, severity is commonly evaluated using global scores such as APACHE and SOFA. Knaus et al. developed the APACHE score as a tool for classifying disease severity in critically ill patients [2], while Vincent et al. introduced the SOFA score to describe the extent of organ dysfunction in the ICU [3]. These scores remain useful for estimating risk and comparing patient groups, but they provide a global measure of severity and may not fully capture the biological mechanisms that influence individual outcomes. Gordon et al. recently emphasized this limitation, showing that critical care research is increasingly moving beyond broad syndromic categories toward sub-phenotypes defined by integrated clinical, physiological, and biological data [4].

In recent years, critical care research has focused increasingly on distinct clinical and biological phenotypes. This approach reflects the observation that patients with the same diagnosis or similar severity scores may have different pathophysiological mechanisms and, consequently, different clinical outcomes. ICU sub-phenotyping is, therefore, viewed as an important step toward precision medicine, with potential value for risk stratification, trial enrichment, and the interpretation of heterogeneous treatment responses [4,5].

In sepsis, for example, coagulation activation is recognized as an early and clinically relevant component of disease progression. Sepsis-induced coagulopathy reflects the interaction between systemic inflammation, endothelial activation, platelet dysfunction, and hemostatic imbalance, while progression to disseminated intravascular coagulation is associated with poor prognosis [6]. Recent studies also suggest that classical coagulation markers, such as D-dimer and INR, should be interpreted together with inflammatory markers and the overall clinical context, rather than as isolated variables [7].

This link between inflammation and coagulation is partly explained by the concept of immune–thrombosis. In critical illness, endothelial dysfunction, glycocalyx degradation, leukocyte and platelet activation, and microthrombus formation may contribute to tissue hypoperfusion and organ dysfunction [8]. Thus, inflammatory markers, coagulation parameters, and markers of tissue injury may reflect different components of the same systemic physiological process.

Respiratory impairment is another important dimension of critical illness. Hypoxemia, changes in arterial carbon dioxide, and acid–base disturbances may reflect not only pulmonary dysfunction but also overall disease severity, impaired gas exchange, altered oxygen delivery, and compensatory metabolic responses. Biological phenotyping in sepsis and acute respiratory distress syndrome may help distinguish patients who appear clinically similar but differ in inflammatory profile, respiratory involvement, and outcome [9]. In ARDS, previous phenotypic analyses have identified biologically distinct subgroups, including a hyperinflammatory phenotype associated with more severe inflammation, metabolic acidosis, shock, and higher mortality [10,11,12]. Further studies have shown that these sub-phenotypes can be recognized using clinical and biological variables routinely available in the intensive care unit, supporting the utility of phenotyping in stratifying critically ill patients [13,14].

A similar approach has been applied in sepsis, where distinct clinical phenotypes have been associated with differences in biological profiles, organ dysfunction, host response, and mortality [15]. Recent reviews also highlight the clinical relevance of sepsis sub-phenotypes, particularly for identifying patients with different diagnostic or prognostic profiles, as well as potential differences in treatment response [5]. This supports the concept that critical illness should be viewed as a spectrum of physiological states rather than as a single uniform condition. In this setting, multimarker assessment may provide a more informative evaluation of risk than isolated biomarkers.

Comatose patients admitted to the intensive care unit represent a particular subgroup within the critically ill population. Although coma is primarily defined by a profound impairment in consciousness and is often approached from a neurological perspective, patient outcome may also be influenced by systemic extracerebral processes. Robba et al. showed that abnormalities in systemic oxygenation are associated with outcome in patients with acute brain injury, supporting the clinical relevance of respiratory and gasometric variables in neurologically vulnerable ICU patients [16]. Similarly, Yao et al. emphasized the interaction between extracerebral organ dysfunction and brain injury, highlighting the bidirectional relationship between systemic failure and neurological outcome [17]. Inflammation, coagulopathy, hypoxemia, acid–base disturbances, and tissue injury may, therefore, coexist at ICU admission and contribute to prognosis alongside neurological severity. Recent work on acute brain dysfunction in the ICU has suggested that coma and delirium can also be examined through data-driven phenotyping, reflecting the dynamic and heterogeneous nature of neurological dysfunction in critical illness [18]. However, data on the physiological phenotypes of critically ill comatose patients remain limited.

Based on this premise, the present study aimed to analyze the relationships between inflammatory markers, coagulation parameters, tissue injury markers, respiratory variables, acid–base parameters and clinical severity scores in comatose patients admitted to intensive care. In addition, the study aimed to identify physiological phenotypes through cluster analysis and evaluate their association with ICU outcome. The main hypothesis was that critically ill comatose patients do not constitute a biologically homogeneous population but present distinct multidimensional physiological profiles associated with different mortality patterns.

## 2. Materials and Methods

### 2.1. Study Design

A retrospective observational study was conducted on a cohort of comatose patients admitted to the Anesthesia and Intensive Care Unit of the “Sf. Apostol Andrei” County Emergency Clinical Hospital, Galați, between December 2025 and March 2026, following ethical approval (no. 26370, approval date: 24 November 2025).

The analysis was based on data previously recorded in the patients’ medical records, without additional interventions and without changing the diagnostic or therapeutic conduct. The information was extracted from the observation sheets, intensive care records, laboratory test results and documents regarding the clinical evolution.

### 2.2. Participants and Selection Criteria

The presence of coma was established retrospectively, based on the records from the medical documents at admission, including the initial clinical assessment, description of the neurological status and the attending physician’s mentions of coma or comatose state. As this was a retrospective study, Glasgow Coma Scale scores were not consistently documented in medical records and could not be applied as a standardized measure across all cases. Coma status was instead determined based on clinical assessment recorded upon admission to the ICU, rather than a uniform numerical score.

From the initial cohort of 324 patients, 97 were excluded, so the final group consisted of 227 patients admitted to the intensive care unit. Adult patients admitted to the ICU in a comatose state at admission, for whom the necessary data for the proposed analyses were available, were considered eligible. These data included demographic data, information on invasive mechanical ventilation, biological and gasometric parameters, APACHE II and SOFA scores and evolution in intensive care, defined as death in the ICU or transfer to the ward.

Patients without coma at ICU admission, cases with incomplete medical documentation, patients whose ICU outcome could not be established, and those lacking essential biological or blood gas data for correlation analysis or cluster analysis were excluded. In the case of isolated missing values, patients were not eliminated from the entire cohort, but only from the analysis of the respective variable.

Invasive mechanical ventilation was required in 68.3% of patients (155/227), predominantly volume-controlled ventilation (97 patients), with BiPAP, APRV or combined modes used in the remainder; this overlap between coma and the need for airway protection should be considered when interpreting the cohort.

Data on sedation status or dosage were not available in these records, for either ventilated or non-ventilated patients; therefore, it could not be determined whether any patient received sedation or not.

### 2.3. Data Collection

Data were collected by retrospectively reviewing the available medical records for each patient included in the study. Information recorded at the time of admission to intensive care or during the first biological and gasometric assessments performed after admission was used.

Age, sex, need for invasive mechanical ventilation, APACHE II score, SOFA score, and ICU outcome were recorded for each patient. Outcome was classified into two categories: death in intensive care or transfer to the ward.

Parameters analyzed at ICU admission included C-reactive protein, procalcitonin, D-dimers, INR, lactate dehydrogenase, peripheral oxygen saturation, arterial oxygen, arterial carbon dioxide, and arterial bicarbonate. These parameters were selected because they reflect processes relevant to critical illness, such as systemic inflammation, coagulation activation, tissue damage, oxygenation impairment, ventilatory status, and acid–base changes.

The analysis used values obtained at admission to intensive care or values closest to the time of admission when multiple measurements were available.

Blood gas and laboratory samples were drawn at ICU admission, representing the first set of results available after the patient came in. The dataset also included measurements taken at 24 h and at ICU discharge, but these were not used in the analyses reported here. APACHE II I and SOFA scores were both calculated from data collected in the first 24 h of admission. Given the retrospective design, we could not establish with certainty, case by case, whether samples were drawn before or after initial interventions such as mechanical ventilation, oxygen therapy, vasopressors, or resuscitation.

The circumstances of death (cardiorespiratory arrest versus a decision to limit or withdraw life-sustaining therapy) were not captured as a structured variable in the database. Based on clinical knowledge of the cases, however, most ICU deaths in this cohort resulted from cardiorespiratory arrest or treatment failure despite maximal active care, while decisions to limit or withdraw life-sustaining therapy were uncommon in this population.

### 2.4. Variables and Physiological Domains Analyzed

The variables included in the analysis were organized into physiological domains to facilitate the interpretation of the results. The inflammatory domain included CRP and procalcitonin, and the coagulation domain included D-dimers and INR. LDH was used as a marker of tissue damage. It is a nonspecific enzyme released from many different cell types, so elevated values do not point to injury in any one particular organ. The respiratory and acid–base domain included SpO_2_, PaO_2_, PaCO_2_ and arterial bicarbonate.

The APACHE II and SOFA scores were analyzed as indicators of global clinical severity. They were included to assess whether the phenotypes identified by cluster analysis only reflect the overall severity of disease or whether they highlight distinct biological profiles that are not fully captured by conventional scores.

### 2.5. Statistical Analysis

Statistical analysis was performed using IBM SPSS Statistics, version 28 (IBM Corp., Armonk, NY, USA). The statistical analysis was performed based on the data available for the patients included in the cohort. The distribution of continuous variables was assessed before applying statistical tests. Since most of the analyzed parameters did not have a normal distribution, continuous variables are presented as median and interquartile range, and categorical variables as number and percentage.

The relationships between biological markers, respiratory parameters, acid–base values and severity scores were evaluated by Spearman correlation. Statistically significant correlations are reported by the Spearman rho coefficient and *p*-values. Because many pairwise correlations were tested, we applied Benjamini–Hochberg correction for the false discovery rate, and both raw and adjusted *p*-values were calculated.

Hierarchical cluster analysis was used to identify physiological phenotypes. The variables included in the analysis were previously standardized as z-scores to reduce the influence of scale differences between the parameters. The nine variables that went into the clustering were CRP, procalcitonin, D-dimer, INR, LDH, SpO_2_, PaO_2_, PaCO_2_, and arterial bicarbonate. APACHE and SOFA scores were kept out of the clustering algorithm on purpose; we looked at them afterward, separately, as an independent check on clinical severity. None of the variables used in these analyses had any missing values, as patients with incomplete essential biological or physiological data had already been excluded at the selection stage. So, cluster membership came from complete data, with no need for imputation. Clustering was performed by the Ward method, using the squared Euclidean distance. The final number of clusters was determined based on the dendrogram, the separation between groups, and the clinical interpretability of the obtained profiles.

After establishing the clusters, patient characteristics were compared between groups. The Kruskal–Wallis test was used for continuous variables, and the chi-square test for categorical variables. In the analyses involving the smallest cluster, the results were interpreted with caution, given the low frequencies.

The association between physiological phenotype and ICU outcome was assessed by comparing the proportions of death and transfer per ward between the three clusters. The statistical significance threshold was set at *p* < 0.05, two-sided.

Effect sizes were also calculated. For comparisons made using the Kruskal–Wallis test, epsilon-squared was used to estimate the amplitude of differences between clusters. For chi-square analyses, the intensity of the association between categorical variables was assessed using Cramer’s V.

Cluster stability was assessed by silhouette analysis and bootstrap stability based on the Jaccard index. The silhouette score was used to assess the degree of separation between clusters, with higher values indicating better delimitation between groups. Bootstrap resampling was used to estimate the reproducibility of cluster membership, and Jaccard similarity was calculated for each cluster. The number of clusters was not established solely from the dendrogram. We also calculated the average silhouette width, the Calinski–Harabasz and Davies–Bouldin indices, and the gap statistic (with 50 bootstrap replicates) for k = 2 to k = 8, using the same standardized variables and Ward linkage.

To assess whether physiological phenotype was independently associated with ICU mortality, a logistic regression analysis was performed, with death in intensive care as the dependent variable. Cluster membership was entered as the main predictor, with Cluster 2 used as the reference group. The model was adjusted for age, sex, invasive mechanical ventilation, APACHE II score, and SOFA score. Penalized logistic regression was used to reduce the bias associated with complete separation, specifically Firth’s method with penalized maximum likelihood reduced by bias. Multicollinearity among predictors was assessed using variance inflation factors and the overall condition index.

Sensitivity analyses were performed to evaluate the robustness of the cluster solution, including clustering on log-transformed variables (for CRP, procalcitonin, D-dimer, and LDH, given their skewed distributions) and alternative clustering methods (complete linkage, average linkage, and k-means), compared to the primary solution using the Adjusted Rand Index.

The results are reported as odds ratios, with 95% confidence intervals and *p*-values.

### 2.6. Ethical Considerations

The study was conducted in accordance with the principles of the Declaration of Helsinki and the institutional rules regarding the retrospective use of medical data. The study protocol was approved by the Institutional Ethics Committee, with approval number 26370, approval date: 24 November 2025.

Given the retrospective nature of the study and the use of anonymized data from existing medical documents, individual informed consent was waived by the Ethics Committee. The confidentiality of patient data was maintained throughout the collection, processing and analysis.

## 3. Results

### 3.1. General Characteristics of the Cohort

The final analysis included 227 comatose patients admitted to the ICU. The median age of the cohort was 72 years, and the group included 130 men (57.3%) and 97 women (42.7%).

At admission to the ICU, patients presented with significant oxygenation impairment. The median SpO_2_ value was 85%, and the median arterial oxygen value was 59.0. PaCO_2_ had a median value of 33.2, and arterial bicarbonate had a median value of 22.0. In the context of respiratory impairment, 155 patients (68.3%) required invasive mechanical ventilation.

Biological markers indicated the presence of an inflammatory response and tissue damage. Median values were 96.0 for CRP, 0.76 for procalcitonin, 3.50 for D-dimer, 1.30 for INR and 1151.0 for LDH. Clinical severity was reflected by a median APACHE II score of 17 and a median SOFA score of 7. Regarding ICU outcome, 149 patients (65.6%) died and 78 patients (34.4%) were transferred to the ward (Table 1).

### 3.2. Physiological Network Interactions

Spearman analysis revealed significant correlations between inflammatory markers, coagulation parameters, LDH and respiratory variables. For a clearer presentation, the correlations were grouped into three main physiological domains: the inflammation–coagulation relationship, the hypoxia–tissue damage relationship and the respiratory–acid–base relationship.

#### 3.2.1. Inflammation–Coagulation Relationship

Significant correlations were observed between inflammatory markers and coagulation parameters. CRP was positively correlated with D-dimers (ρ = 0.279; *p* < 0.001), indicating an association between the systemic inflammatory response and coagulation activation, but also with procalcitonin (ρ = 0.243; *p* < 0.001), consistent with concordant behavior among the inflammatory markers analyzed. Procalcitonin was positively correlated with INR (ρ = 0.265; *p* < 0.001), and D-dimers with INR (ρ = 0.291; *p* < 0.001). These correlations are statistically significant, but the coefficients (ρ = 0.243–0.291) fall into the weak range. They indicate a consistent trend for inflammation and coagulation to move together in this cohort, rather than a strong linear relationship.

#### 3.2.2. Hypoxia–Tissue Damage Interactions

LDH showed significant correlations with both oxygenation parameters and inflammatory and coagulation markers. LDH values were negatively correlated with PaO_2_ (ρ = −0.311; *p* < 0.001) and with SpO_2_ (ρ = −0.242; *p* < 0.001). The most pronounced relationship in this group of correlations was that between LDH and arterial PaO_2_, indicating an association between reduced oxygenation and increased tissue damage marker. At the same time, LDH was positively correlated with D-dimers (ρ = 0.387; *p* < 0.001), CRP (ρ = 0.278; *p* < 0.001) and INR (ρ = 0.210; *p* = 0.002). These correlations range from weak to weak-moderate (ρ = 0.210–0.387) and, although statistically significant, should be read as a modest tendency linking tissue damage to impaired oxygenation, inflammation and coagulation, rather than a strong association.

#### 3.2.3. Relationship Between Respiratory Parameters and Acid–Base Balance

In the analysis of respiratory parameters, SpO_2_ tracked closely with PaO_2_ (ρ = 0.351; *p* < 0.001), consistent with the two measures reflecting the same underlying process, when it comes to oxygenation. SpO_2_ also correlated negatively with PaCO_2_ (ρ = −0.181; *p* = 0.006), and PaCO_2_ correlated positively with bicarbonate (ρ = 0.238; *p* < 0.001)—a pattern that fits the acid–base shifts typically seen in critically ill patients. Coagulation and oxygenation also showed a relationship: D-dimers correlated negatively with both PaO_2_ (ρ = −0.185; *p* = 0.005) and SpO_2_ (ρ = −0.178; *p* = 0.007), and INR correlated negatively with PaO_2_ (ρ = −0.150; *p* = 0.024), though this last association did not hold up after FDR correction (Table 2). Taken together, these results suggest that coagulation changes tracked with lower arterial oxygenation, at least for the associations that survived correction. As with the other correlations reported here, the coefficients involved were weak (ρ ≤ 0.185), so this pattern should be read as a modest, statistically detectable tendency rather than a strong relationship.

The main significant physiological correlations are presented in Table 2. We tested 36 pairwise correlations in total; 19 were significant at the raw *p* < 0.05 threshold, and 15 of these remained significant after Benjamini–Hochberg correction. Four borderline associations, INR with PaO_2_, PaO_2_ with bicarbonate, procalcitonin with PaCO_2_, and procalcitonin with D-dimers, did not survive the adjustment.

### 3.3. Physiological Phenotype Identification

To identify physiological subgroups, cluster analysis was performed using inflammatory, coagulation, tissue-injury, respiratory, and acid–base variables. APACHE II and SOFA scores were not included in the clustering algorithm and were evaluated separately after cluster formation. Before analysis, variables were standardized to reduce the influence of scale differences between the included parameters.

Ward hierarchical analysis, using squared Euclidean distance, highlighted three main physiological phenotypes in the analyzed cohort (Supplementary Figure S1). The distribution of patients between clusters was uneven: Cluster 1 included 61 patients (26.9%), Cluster 2 included 155 patients (68.3%), and Cluster 3 was the smallest, with 11 patients (4.8%).

Cluster 1 had a profile characterized by higher values of CRP, D-dimers, INR and LDH, compared to Cluster 2. At the same time, PaO_2_ and SpO_2_ values were lower.

Cluster 2 was the largest and generally had the lowest values of inflammatory, coagulation, and tissue damage markers. This group had a less altered biological profile than the other two clusters.

Cluster 3, although it included a small number of patients, was distinguished by the highest values of CRP and procalcitonin. D-dimers and LDH were also increased, and PaO_2_ had the lowest values among the three groups. Higher values of PaCO_2_ and bicarbonate were also observed in this cluster (Supplementary Figure S2).

The Kruskal–Wallis test showed significant differences between clusters for CRP, procalcitonin, D-dimers, INR, LDH, PaCO_2_ and arterial bicarbonate. In contrast, SpO_2_, APACHE II and SOFA did not differ significantly between clusters. These results indicate that the separation of phenotypes was mainly determined by biological markers and blood gas parameters, rather than by classical clinical scores (Table 3).

Although Cluster 3 had the highest mortality and important biological changes, it did not have the highest median APACHE II or SOFA scores. This result suggests that physiological phenotypes may capture biological particularities that are not fully reflected by conventional clinical scores.

To complement the results of the statistical significance tests, effect sizes were also calculated. For comparisons between the three clusters performed using the Kruskal–Wallis test, epsilon-squared (ε^2^) was used. The highest ε^2^ values were observed for D-dimers, CRP, procalcitonin, INR and LDH, indicating that these variables contributed the most to the differentiation of physiological phenotypes. Of these, D-dimers had the largest effect size (ε^2^ = 0.310). Blood gas parameters had smaller effects, and for SpO_2_, APACHE II and SOFA, the effects were small or negligible (Table 4).

### 3.4. Post Hoc Analysis of Between-Cluster Differences

The results of the post hoc analysis show that the differences between Cluster 1 and Cluster 2 are mainly explained by the values of CRP, procalcitonin, D-dimers, INR and LDH. Thus, Cluster 1 emerges as a group with more important inflammatory and coagulopathic activation, associated with higher values of the tissue damage marker, compared to Cluster 2.

Cluster 3 is differentiated mainly by the values of procalcitonin and by the gasometric parameters, including arterial PaO_2_, PaCO_2_ and arterial bicarbonate. This profile suggests a subgroup with more pronounced respiratory and acid–base impairment, against the background of a significant inflammatory response. The fact that CRP and D-dimers were significantly different between Cluster 2 and Cluster 3, but not between Cluster 1 and Cluster 3, indicates that this cluster does not simply represent a more severe overall form of the other groups, but a distinct physiological profile (Table 5).

No significant overall differences were identified for SpO_2_, APACHE II, and SOFA by the Kruskal–Wallis test. For this reason, these variables were not included in the main post hoc interpretation.

### 3.5. Internal Validation of Cluster Stability

The stability of the clusters was verified by silhouette analysis and by estimating the bootstrap stability based on the Jaccard index. The overall silhouette score was 0.172, which shows a modest separation between the three phenotypes. Cluster 1 had the lowest silhouette values, suggesting a partial overlap with the other groups. In contrast, Cluster 2 and Cluster 3 had higher values, indicating a relatively better delineation in the multidimensional space. The bootstrap Jaccard values obtained were 0.242 for Cluster 1, 0.402 for Cluster 2 and 0.457 for Cluster 3, indicating limited to moderate internal stability.

We also tested whether three clusters was really the best choice, by comparing the average silhouette width, the Calinski–Harabasz and Davies–Bouldin indices, and the gap statistic for solutions from k = 2 to k = 8 (Supplementary Table S1). Silhouette width and the Calinski–Harabasz index were both a little higher for two clusters (0.284 and 37.8) than for three (0.252 and 33.9), and the gap statistic did not point to a clearly separated structure at any k, which fits with the modest overall silhouette already reported above. Still, the two-cluster solution turned out to be nested inside the three-cluster one: the larger, less severe group did not change at all, while the more severe group was further split into an intermediate subgroup and the smallest, most biologically altered one (Cluster 3), which also had the highest mortality. Because the statistical evidence for two versus three clusters was so close, and three clusters kept this clinically relevant distinction, we decided to keep the three-cluster solution for the rest of the analysis.

Agreement with the primary Ward solution varied considerably across alternative specifications, from moderate for complete linkage (ARI = 0.731) to very weak for average linkage (ARI = 0.142, which produced a near-degenerate solution) (Supplementary Table S4). Because the mortality rate of Cluster 3 was high, we also examined the two-cluster solution, in which the severe group was expanded to 44 patients by merging with the intermediate subgroup. Mortality in this larger group remained very high, at 95.5% (42/44), although it was no longer absolute. This indicates that the 100% mortality observed in the smaller, three-cluster version of Cluster 3 is not an artifact of a narrowly drawn boundary, but reflects a pattern that persists even as the group size increases.

Therefore, the identified phenotypes should be interpreted as exploratory results, requiring confirmation in independent cohorts (Table 6).

### 3.6. Association Between Physiological Phenotypes and ICU Outcome

ICU outcome differed between the three physiological phenotypes, with the difference being statistically significant (χ^2^ = 18.370; *p* < 0.001). High mortality was also observed in Cluster 1, where 50 deaths out of 61 patients (82.0%) were recorded. Cluster 3 had the most unfavorable evolution, with all 11 patients (100.0%; exact 95% CI, 71.5–100%) dying in the ICU.

Cluster 2 had a more favorable evolution compared to the other two groups. In this cluster, 88 patients (56.8%) died, and 67 patients (43.2%) were transferred to the ward (Table 7).

These data indicate an association between physiological phenotype and clinical evolution in the ICU. The result for Cluster 3 should be interpreted with caution, as this subgroup included a small number of patients.

The association between physiological phenotype and outcome in intensive care was statistically significant (χ^2^ = 18.370; *p* < 0.001), with a Cramer’s V of 0.284, indicating a low-to-moderate-intensity association.

### 3.7. Adjusted Analysis for ICU Mortality

To test whether belonging to a particular physiological phenotype was independently associated with ICU mortality, an adjusted logistic regression was performed. Cluster 2 was used as the reference group, and the model included age, sex, invasive mechanical ventilation, APACHE II score, and SOFA score. Given that Cluster 3 had 100% mortality, penalized logistic regression was used to limit the effect of complete separation on the estimates.

In the adjusted analysis, cluster membership was not independently associated with ICU mortality. Compared with Cluster 2, patients in Cluster 1 had higher odds of death, but the association was not statistically significant (OR = 2.18; 95% CI: 0.39–12.28; *p* = 0.376). Cluster 3 also did not remain independently associated with mortality after adjustment. The wide confidence interval for this subgroup reflects the small number of patients and the complete mortality observed within the cluster (Table 8). A leave-one-out sensitivity analysis, excluding each of the 11 Cluster 3 patients in turn, showed that the odds ratio for Cluster 3 remained stable (range, 0.86–1.21), indicating that this estimate was not driven by any single case.

Age and invasive mechanical ventilation were associated with ICU mortality. However, the very large odds ratio for invasive mechanical ventilation points to an unstable estimate, consistent with near-complete separation rather than collinearity. Therefore, this result should be interpreted with caution. Given this instability, the adjusted odds ratio for invasive mechanical ventilation should not be taken as something to base clinical decisions on—it is really just a reflection of the near-complete separation in the data, not a precise or generalizable effect estimate. The inverse association with APACHE II score held up across all the reduced models we tried, even the unadjusted one with APACHE II on its own, so model instability or collinearity does not explain it (Supplementary Table S3). The inverse association between APACHE II score and mortality remained consistent across the reduced and unadjusted models. However, because individual Glasgow Coma Scale values and sedation status were not consistently available in the retrospective records, the contribution of the neurological component of APACHE II or of sedation to this unexpected association could not be evaluated. This finding should, therefore, be interpreted cautiously and requires confirmation in independent cohorts.

VIF values stayed between 1.06 and 1.48 for every predictor, and the condition index came out at 2.03. So, multicollinearity is not behind the huge odds ratio for invasive mechanical ventilation. What actually explains it is much simpler: almost no one survived without ventilation and almost no one died when it stayed off the table as an outcome-changer; only 2.8% of non-ventilated patients died, versus 94.8% of those who were ventilated (Supplementary Table S2).

Given the instability of the full model, a simplified model excluding invasive mechanical ventilation was also fitted; all confidence intervals in this reduced model remained within a clinically interpretable range (Supplementary Table S5).

Since the inverse association between APACHE II score and mortality was unexpected, we tested it under several reduced models, dropping invasive mechanical ventilation, dropping SOFA, and finally testing an unadjusted univariable model with APACHE II alone. The odds ratio barely moved across these models (0.92 to 0.95), even without any adjustment at all, which argues against model instability or collinearity as the explanation. In fact, at the descriptive level, the pattern was the same: median APACHE II was higher in survivors (24) than in patients who died in the ICU (16; *p* < 0.001, Mann–Whitney U test) (Supplementary Table S3).

## 4. Discussion

The present study suggests that comatose patients admitted to the intensive care unit are not biologically uniform. Despite the common clinical feature of severely impaired consciousness, the cohort showed relevant differences in inflammatory, coagulation, respiratory, acid–base and tissue-injury profiles. Correlation analysis outlined three main biological axes: inflammation–coagulation, hypoxia–tissue injury and respiratory–acid–base imbalance. Cluster analysis further suggested three tentative physiological phenotypes: an inflammatory–coagulopathic phenotype, a relatively stable phenotype and a severe respiratory–inflammatory phenotype, though, as detailed below, internal validation indicated only modest separation between these groups. These phenotypes were associated with different ICU outcomes, suggesting that prognosis in comatose ICU patients may depend not only on global severity, but also on the interaction between several systemic processes.

This is consistent with recent work showing that combining several routine physiological and laboratory parameters does a better job of early risk stratification in critically ill older adults than any single biomarker or variable on its own [19,20], which is really the same rationale behind the multidimensional phenotyping approach we used here.

This finding fits with the current move toward precision medicine in critical care, where broad ICU syndromes are increasingly viewed as heterogeneous conditions rather than single entities. Recent recommendations on ICU sub-phenotyping emphasize that biological and clinical subgroups may help explain differences in prognosis, disease evolution and treatment response [21]. Similarly, studies based on early physiological signatures have shown that clusters of critically ill patients may identify distinct clinical trajectories without simply reproducing established severity scores [22]. In this context, the phenotypes described in our cohort should be considered exploratory biological profiles that may add information to standard clinical assessment.

One of the main findings was how closely inflammation and coagulation tracked together: CRP correlated with D-dimers, procalcitonin with INR, and D-dimers with INR, suggesting that these two processes developed hand in hand in this cohort. This lines up with the concept of immunothrombosis, endothelial injury, glycocalyx damage, leukocyte–platelet adhesion and coagulation activation all linking inflammation to thrombosis in sepsis and critical illness [23,24,25,26,27]. None of this proves causality, but it does show that inflammation and coagulation were not acting as separate processes here, and it means coagulation markers carry more weight when read alongside inflammatory biomarkers and the overall clinical picture [7,25].

Cluster 1 showed this pattern most consistently, with higher CRP, D-dimers, INR and LDH, along with higher unadjusted mortality, which puts it close to the thrombo-inflammatory profiles seen in critically ill patients, where coagulation problems can range anywhere from thrombotic to hemorrhagic [28].

Another key finding was how LDH tied together with hypoxia and the inflammatory–coagulopathic markers: it correlated negatively with PaO_2_ and SpO_2_ and positively with CRP, D-dimers and INR, suggesting that tissue injury does not happen in isolation, but alongside impaired oxygenation. This lines up with reports that the LDH-to-albumin ratio predicts mortality in ARDS [29]. LDH is nonspecific: in critically ill patients, it can reflect cellular hypoxia, hepatic injury, hemolysis, generalized tissue damage, or multiorgan dysfunction, either alone or in combination. This differs from the organ-specific injury markers used elsewhere in critical care, such as troponin for myocardial injury or transaminases for hepatic injury, which allow damage to be localized to a specific organ. Since no such organ-specific markers were available in this study, the “tissue-injury” component of the phenotypes described here should be interpreted as a signal of nonspecific cellular stress rather than injury confined to a particular organ system.

D-dimers’ negative correlation with PaO_2_ points to something similar, coagulation activation overlapping with impaired oxygenation, maybe through pulmonary microthrombosis or endothelial dysfunction, though this is only an association, not proof that D-dimers themselves drive hypoxemia. Combined indices like the D-dimer-to-albumin ratio have been tied to short-term mortality in sepsis [30,31], and admission D-dimer levels to septic shock in recent cohorts [32].

The respiratory and acid–base side of things showed up mainly as a positive correlation between PaCO_2_ and bicarbonate, which fits with the compensatory mechanisms in respiratory dysfunction, plus the expected agreement between SpO_2_ and PaO_2_. This mattered most for Cluster 3, where respiratory abnormalities, a sharp rise in procalcitonin, and the worst ICU outcome (100% mortality) all came together, a pattern that looks a lot like the hyperinflammatory ARDS sub-phenotypes described elsewhere, consistently tied to stronger inflammation, shock, metabolic acidosis, and worse outcomes in studies using routine variables and machine learning approaches [13,14,33,34,35]. Our biomarker panel is not the same as these ARDS latent-class analyses, but the direction is: patients with heavy inflammation and respiratory impairment did worst.

The marked procalcitonin elevation in Cluster 3 may indicate a more intense infectious or inflammatory burden, though this interpretation is limited by the retrospective design and the absence of detailed data on infection source, microbiology and antimicrobial exposure, variables that future phenotyping models incorporating healthcare-associated infection and resistance patterns should include [36].

The mortality observed in Cluster 3 should nevertheless be interpreted carefully. This subgroup included only 11 patients, and all died in the ICU. This complete separation of outcome limits the reliability of conventional logistic regression estimates and makes standard odds ratios unstable, though a leave-one-out check showed that this instability was not coming from any single patient. Therefore, the association between Cluster 3 and mortality should be regarded as an exploratory signal, not as a validated independent prognostic effect. Larger cohorts and penalized logistic regression approaches would be needed to confirm this finding.

Cluster 2 had the most stable biological profile and the most favorable outcome. Lower inflammatory, coagulation and tissue-injury marker values suggest less intense systemic activation. This is consistent with sepsis phenotyping research showing that critically ill patients can be grouped into clinically meaningful phenotypes with different host-response patterns, organ dysfunction profiles and mortality risks [37]. In this sense, Cluster 2 may represent a lower-biological-risk phenotype within a generally high-risk comatose ICU population.

An important finding of this study is the discrepancy between physiological phenotypes and classical severity scores. APACHE II showed only a borderline, non-significant difference between clusters, while SOFA did not differ significantly. Moreover, Cluster 3 had the highest mortality despite not having the highest median APACHE II or SOFA scores. This suggests that conventional scores may miss some high-risk biological profiles, particularly when risk is driven by the interaction between inflammation, hypoxia, coagulation activation and tissue injury. Similar observations have been reported in acute illness phenotyping studies, where early physiological clusters did not simply mirror existing severity scores [17]. Time-updated modeling in comatose ICU patients also supports the added value of dynamic physiological information beyond static baseline assessment [35].

None of this diminishes APACHE II or SOFA; they are still useful for gauging overall severity and comparing ICU populations. But our results suggest that multidimensional profiling can add something on top: two patients with similar APACHE II or SOFA scores might still carry different risk depending on whether they show an inflammatory–coagulopathic or a respiratory pattern, which fits evidence that persistent coagulopathy and rising SOFA scores predict mortality in sepsis [38]. More broadly, CRP, procalcitonin, D-dimers, INR, LDH and the blood gas parameters seemed to matter more together than any one of them alone, echoing recent calls for multimarker strategies over single biomarkers in critical illness [39,40].

The clinical implications should remain cautiously interpreted. The present findings do not support treatment decisions based solely on cluster membership. However, they suggest that patients with inflammatory–coagulopathic or respiratory–inflammatory profiles may require closer monitoring of coagulation status, repeated reassessment of oxygenation, follow-up of tissue-injury markers and careful evaluation of infectious or inflammatory sources. These implications should be considered hypothesis-generating rather than practice-changing.

Internal validation showed modest separation between phenotypes. The overall silhouette score was low (0.172), and bootstrap Jaccard values (0.242–0.457) indicated only limited-to-moderate cluster stability. Given these figures, the three phenotypes described here should be read primarily as exploratory, data-driven groupings rather than as confirmed, biologically robust subtypes. This does not invalidate the biological relevance of the profiles, but it confirms the exploratory nature of the analysis. Current methodological recommendations for ICU sub-phenotype research emphasize transparent reporting, internal validation and external replication before exploratory phenotypes can be considered clinically actionable [4]. Therefore, the clusters described here should be interpreted as data-derived physiological profiles requiring external validation.

### Study Limitations

The study has several limitations. First, the identified correlations do not establish causal relationships. Second, the cluster analysis is exploratory in nature and requires external validation. Third, coma was identified retrospectively, rather than by a standardized scale such as the GCS, and sedation could not be taken into account, which limits the precision with which “coma” is defined in this cohort. Fourth, Cluster 3 includes a small number of patients, which requires caution in interpreting the 100% mortality. The exact 95% confidence interval for this estimate was wide (71.5–100%), and a leave-one-out analysis showed that the odds ratio stayed within a narrow range (0.86–1.21) regardless of which patient was excluded. So, the finding does not depend on a single case, but the small sample still leaves real uncertainty around it.

One important limitation is that we did not have a structured way to classify what actually caused the coma in each patient. This was a general ICU cohort, and the underlying cause (traumatic brain injury, ischemic or hemorrhagic stroke, sepsis-associated encephalopathy, toxic–metabolic causes, post-cardiac arrest encephalopathy, and so on) was not systematically recorded. These are biologically very different conditions, so we cannot rule out that the clusters we found partly reflect differences in diagnosis rather than a physiological pattern that holds regardless of cause. As a result, coma etiology could not be reported either for the overall cohort or broken down by cluster, and this remains an open question for future studies with more detailed etiological data. A few other variables were also missing from this analysis, including septic status, anticoagulant treatment, vasopressor use, renal function, and the exact timing of sample collection. These are not just incidental gaps—renal function and vasopressor requirement, in particular, can independently shape both the biomarkers analyzed here and ICU mortality, so their absence leaves some residual confounding that this analysis simply cannot account for. Although an adjusted logistic analysis was performed, its interpretation is limited by the small size of Cluster 3, the complete mortality observed in this subgroup and the risk of statistical separation. In addition, the modest internal stability of the clusters supports the need for external validation.

Most deaths in this cohort resulted from cardiorespiratory arrest under maximal treatment, with therapy limitation decisions being rare, but the circumstances of death were not recorded as a structured variable. This observation is based on clinical knowledge of the cases rather than actual data, so it could not be classified case by case.

On top of that, this study only looked at ICU discharge status as the outcome—we did not have follow-up data past that point, so hospital mortality or death at 28 or 30 days could not be assessed. That means our conclusions are limited to the ICU stay itself; some patients transferred to the ward may still have died during the same hospitalization, or shortly after, and we simply do not know. Future studies with longer follow-up would be needed to see whether these physiological phenotypes actually predict outcomes beyond the ICU.

## 5. Conclusions

The present study suggests that comatose patients admitted to intensive care can be tentatively grouped into exploratory physiological phenotypes, associated with different clinical outcomes. The inflammatory–coagulopathic phenotype and the severe respiratory–inflammatory phenotype were associated with higher mortality in the unadjusted analysis, while the relatively stable phenotype had the most favorable clinical outcome. However, after adjustment for relevant clinical factors, cluster membership did not remain an independent predictor of mortality, suggesting that the relationship between phenotype and outcome is influenced by clinical severity and the need for ventilatory support.

These results support the utility of a multidimensional biological assessment, integrating inflammatory markers, coagulation parameters, tissue damage markers, and blood gas variables. The identified phenotypes should be interpreted as exploratory results, with hypothesis-generating value, and require external validation in independent cohorts before they can be used for clinical stratification or phenotype-guided therapeutic decisions.

## Figures and Tables

**Table 1:** Baseline characteristics of the study cohort.

Variable	Total Cohort, *n* = 227
Age, years	72 [16.0]
Male sex, *n* (%)	130 (57.3%)
Female sex, *n* (%)	97 (42.7%)
Invasive mechanical ventilation, *n* (%)	155 (68.3%)
No invasive mechanical ventilation, *n* (%)	72 (31.7%)
SpO_2_ at ICU admission, %	85 [11.0]
PaO_2_ at ICU admission (mmHg)	59.0 [19.7]
PaCO_2_ at ICU admission (mmHg)	33.2 [8.8]
Arterial bicarbonate at ICU admission (mmol/L)	22.0 [8.5]
CRP at ICU admission (mg/L)	96.0 [106.0]
Procalcitonin at ICU admission (ng/mL)	0.76 [2.54]
D-dimers at ICU admission (µg/mL FEU)	3.50 [1.50]
INR at ICU admission	1.30 [0.50]
LDH at ICU admission (U/L)	1151.0 [1118.75]
APACHE II score	17 [16.0]
SOFA score	7 [5.0]
ICU death, *n* (%)	149 (65.6%)
Transfer to ward, *n* (%)	78 (34.4%)

Data are presented as median [interquartile range] or *n* (%).

**Table 2:** Significant physiological correlations in comatose ICU patients.

Physiological Interaction	Spearman ρ	*p* Value (Raw)	*p* Value (FDR-Adjusted)	Significant After FDR
D-dimers ↔ LDH	0.419	<0.001	<0.001	Yes
SpO_2_ ↔ PaO_2_	0.351	<0.001	<0.001	Yes
CRP ↔ LDH	0.324	<0.001	<0.001	Yes
D-dimers ↔ INR	0.319	<0.001	<0.001	Yes
LDH ↔ PaO_2_	−0.311	<0.001	<0.001	Yes
CRP ↔ D-dimer	0.307	<0.001	<0.001	Yes
Procalcitonin ↔ INR	0.294	<0.001	<0.001	Yes
CRP ↔ Procalcitonin	0.272	<0.001	<0.001	Yes
PaCO_2_ ↔ Bicarbonate	0.238	<0.001	0.001	Yes
LDH ↔ SpO_2_	−0.235	<0.001	0.001	Yes
INR ↔ LDH	0.217	0.001	0.003	Yes
D-dimers ↔ PaO_2_	−0.185	0.005	0.016	Yes
SpO_2_ ↔ PaCO_2_	−0.181	0.006	0.018	Yes
D-dimer ↔ SpO_2_	−0.178	0.007	0.018	Yes
CRP ↔ PaCO_2_	0.175	0.008	0.020	Yes
INR ↔ PaO_2_	−0.150	0.024	0.054	No
PaO_2_ ↔ Bicarbonate	−0.139	0.036	0.076	No
Procalcitonin ↔ PaCO_2_	0.137	0.040	0.079	No
Procalcitonin ↔ D-dimers	0.135	0.042	0.080	No

**Table 3:** Physiological characteristics according to cluster membership.

Variable	Cluster 1, *n* = 61	Cluster 2, *n* = 155	Cluster 3, *n* = 11	*p* Value
CRP at ICU admission (mg/L)	156.0 [100.9]	80.0 [76.0]	234.0 [35.3]	<0.001
Procalcitonin at ICU admission (ng/mL)	1.50 [3.41]	0.28 [1.58]	32.00 [13.00]	<0.001
D-dimers at ICU admission (µg/mL FEU)	4.50 [1.90]	3.20 [1.10]	5.30 [2.60]	<0.001
INR at ICU admission	1.67 [0.60]	1.21 [0.31]	1.62 [0.42]	<0.001
LDH at ICU admission (U/L)	1769.0 [1380.5]	982.0 [1052.0]	2162.0 [1790.0]	<0.001
SpO_2_ at ICU admission, %	83.0 [13.0]	85.5 [11.0]	85.0 [7.0]	0.063
PaO_2_ at ICU admission (mmHg)	55.6 [15.3]	61.3 [27.4]	53.9 [6.0]	0.003
PaCO_2_ at ICU admission (mmHg)	34.6 [11.0]	32.5 [8.6]	37.7 [40.8]	0.001
Arterial bicarbonate at ICU admission (mmol/L)	21.0 [10.0]	22.8 [7.1]	27.0 [16.6]	0.022
APACHE II score	17.0 [18.0]	18.0 [14.0]	16.0 [9.0]	0.057
SOFA score	8.0 [7.0]	7.0 [5.0]	4.0 [6.0]	0.359

Data are presented as median [interquartile range]. Between-cluster comparisons were performed using the Kruskal–Wallis test. Missing values were excluded from variable-specific analyses. Differences in the nine variables used to build the clusters (CRP through bicarbonate) are expected by design and are reported here descriptively. APACHE and SOFA were not part of the clustering, so they are the only genuinely independent comparison in this table.

**Table 4:** Effect sizes for between-cluster differences based on the Kruskal–Wallis test.

Variable	H	*p*	ε^2^
CRP	57.040	<0.001	0.246
Procalcitonin	49.924	<0.001	0.214
D-dimers	71.482	<0.001	0.310
INR	50.914	<0.001	0.218
LDH	36.882	<0.001	0.156
SpO_2_	5.517	0.063	0.016
PaO_2_	11.477	0.003	0.042
PaCO_2_	14.218	0.001	0.055
Arterial bicarbonate	7.601	0.022	0.025
APACHE II score	5.723	0.057	0.017
SOFA score	2.050	0.359	0.000

Note: Effect size was estimated using epsilon-squared (ε^2^). Larger ε^2^ values indicate greater between-cluster separation for the corresponding variable.

**Table 5:** Dunn post hoc pairwise comparisons with Benjamini–Hochberg correction.

Variable	Comparison	z	*p* Adjusted BH
CRP	C1 vs. C2	6.534	<0.001
CRP	C1 vs. C3	−1.204	0.229
CRP	C2 vs. C3	−4.435	<0.001
Procalcitonin	C1 vs. C2	4.172	<0.001
Procalcitonin	C1 vs. C3	−3.901	<0.001
Procalcitonin	C2 vs. C3	−6.118	<0.001
D-dimers	C1 vs. C2	7.939	<0.001
D-dimers	C1 vs. C3	0.159	0.874
D-dimers	C2 vs. C3	−3.687	<0.001
INR	C1 vs. C2	6.969	<0.001
INR	C1 vs. C3	1.506	0.132
INR	C2 vs. C3	−1.803	0.107
LDH	C1 vs. C2	5.807	<0.001
LDH	C1 vs. C3	1.261	0.207
LDH	C2 vs. C3	−1.500	0.200
PaO_2_	C1 vs. C2	−1.965	0.051
PaO_2_	C1 vs. C3	1.953	0.051
PaO_2_	C2 vs. C3	3.002	0.008
PaCO_2_	C1 vs. C2	3.347	0.002
PaCO_2_	C1 vs. C3	−0.531	0.595
PaCO_2_	C2 vs. C3	−2.179	0.044
Arterial bicarbonate	C1 vs. C2	−1.803	0.071
Arterial bicarbonate	C1 vs. C3	−2.565	0.031
Arterial bicarbonate	C2 vs. C3	−1.820	0.071

**Table 6:** Internal validation of cluster stability.

Cluster	*n*	Mean Silhouette Score	Bootstrap/Jaccard Stability
Cluster 1	61	0.033	0.242
Cluster 2	155	0.221	0.402
Cluster 3	11	0.261	0.457
Overall	227	0.172	0.367

Note: Silhouette scores were calculated using standardized variables included in the clustering analysis. Bootstrap/Jaccard stability was estimated across repeated resampling procedures. Higher values indicate greater cluster stability.

**Table 7:** Association between physiological phenotypes and ICU outcome.

Outcome	Cluster 1, *n* = 61	Cluster 2, *n* = 155	Cluster 3, *n* = 11	χ^2^	*p* Value	Cramer’s V
ICU death, *n* (%)	50 (82.0%)	88 (56.8%)	11 (100.0%)	18.370	<0.001	0.284
Transfer to ward, *n* (%)	11 (18.0%)	67 (43.2%)	0 (0.0%)	—	—	—

Note: Data are presented as *n* (% within cluster). Between-group differences were assessed using the Pearson chi-square test. Cramer’s V was used to estimate the strength of association between physiological phenotype and ICU outcome. One cell had an expected count below 5; therefore, results involving Cluster 3 should be interpreted with caution.

**Table 8:** Adjusted logistic regression analysis for ICU mortality.

Variable	OR	95% CI	*p* Value
Cluster 1 vs. Cluster 2	2.18	0.39–12.28	0.376
Cluster 3 vs. Cluster 2	1.02	0.05–21.75	0.990
Age	1.05	1.01–1.10	0.012
Male gender	1.20	0.30–4.74	0.797
Invasive mechanical ventilation	807.37	110.77–5884.85	<0.001
APACHE II score	0.92	0.85–1.00	0.042
SOFA score	1.22	0.96–1.56	0.104

Note: Cluster 2 was used as the reference category. The outcome variable was ICU death. Penalized logistic regression was used because Cluster 3 had complete mortality and the standard logistic model showed separation.

## Data Availability

The original contributions presented in this study are included in the article/Supplementary Materials. Further inquiries can be directed to the corresponding authors.

## Supplementary Materials

The following supporting information can be downloaded at: https://www.mdpi.com/article/10.3390/diagnostics16172876/s1, Supplementary Figure S1. Dendrogram using Ward Linkage and Supplementary Figure S2. Standardized cluster characteristics heatmap; Supplementary Table S1. Cluster validity indices for k = 2–8 (Ward hierarchical clustering); Supplementary Table S2. Multicollinearity diagnostics for the adjusted logistic regression model; Supplementary Table S3. Sensitivity analysis of the APACHE score odds ratio across alternative model specifications; Supplementary Table S4. Sensitivity analysis: cluster agreement under alternative transformations and clustering methods; Supplementary Table S5. Simplified adjusted model for ICU mortality, excluding invasive mechanical ventilation.

## Abbreviations

The following abbreviations are used in this manuscript:

ICU	Intensive care unit
LDH	Lactate dehydrogenase
APACHE	Acute Physiology and Chronic Health Evaluation
SOFA	Sequential Organ Failure Assessment
D-dimers	Cross-linked fibrin degradation product
CRP	C-reactive protein
INR	International Normalized Ratio
χ^2^	Chi-square
*p*	*p* value
ARDS	Acute respiratory distress syndrome
SpO_2_	Peripheral oxygen saturation
Spearman ρ	Spearman’s rho
*n*	Number of cases
ε^2^	Epsilon-squared
OR	Odds ratio
CI	Confidence interval
PaCO_2_	Partial pressure of arterial carbon dioxide
PaO_2_	Partial pressure of arterial oxygen
