# Supplementary material for: Physiological Phenotypes in Comatose ICU Patients: A Retrospective Multidimensional Analysis
Source: Diagnostics (Basel). 2026 Sep 7;16(17):2876. doi: 10.3390/diagnostics16172876 (PMC13565738; doi:10.3390/diagnostics16172876)
Supplement: Supplementary file 1 [file diagnostics-16-02876-s001.zip › Supplementary Table.pdf]

Supplementary Table 1. Cluster validity indices for k = 2–8 (Ward hierarchical clustering)

k	WCSS	Silhouette (mean)	Calinski-Harabasz	Davies-Bouldin	Gap statistic	SE (gap)
2	1784.6	0.284	37.76	2.066	0.930	0.030
3	1600.2	0.252	33.87	1.673	0.949	0.028
4	1449.8	0.162	32.52	1.674	0.974	0.029
5	1324.1	0.148	31.86	1.824	1.004	0.027
6	1228.4	0.133	30.79	1.747	1.025	0.028
7	1137.8	0.146	30.50	1.596	1.053	0.027
8	1053.4	0.165	30.61	1.544	1.085	0.026

Note: WCSS = within-cluster sum of squares (elbow criterion); Silhouette = average silhouette width; Gap statistic computed using the Tibshirani et al. method with 50 bootstrap reference samples (uniform distribution aligned to the principal components of the data); SE(gap) = standard error of the gap statistic. All indices were computed on the nine standardized physiological variables (CRP, procalcitonin, D-dimer, INR, LDH, arterial O2, arterial CO2, arterial bicarbonate, SpO2) used in the primary cluster analysis.

Supplementary Table 2. Multicollinearity diagnostics for the adjusted logistic regression model

Predictor	Variance Inflation Factor (VIF)
Cluster 1 vs Cluster 2	1.08
Cluster 3 vs Cluster 2	1.10
Age	1.06
Sex	1.09
Invasive mechanical ventilation	1.18
APACHE score	1.48
SOFA score	1.44

Note: Variance inflation factors were calculated for all covariates included in the adjusted logistic regression model (cluster membership, age, sex, invasive mechanical ventilation, APACHE score, SOFA score). The overall condition index for the model was 2.03. VIF values above 5 and condition indices above 30 are generally considered indicative of relevant multicollinearity; none of the predictors approached these thresholds.

Supplementary Table 3. Sensitivity analysis of the APACHE score odds ratio across alternative model specifications

Model specification	OR (APACHE)	95% CI
Full model (cluster + age + sex + IMV + APACHE + SOFA)	0.92	0.86–0.99
Without invasive mechanical ventilation	0.94	0.91–0.98
Without SOFA score	0.95	0.90–1.02
Without IMV and SOFA	0.94	0.91–0.97
Cluster membership + APACHE only	0.94	0.91–0.97
APACHE score alone (unadjusted, univariable)	0.94	0.91–0.97

Note: Odds ratios for APACHE score (per 1-point increase) were estimated using penalized (Firth) logistic regression across reduced model specifications. IMV = invasive mechanical ventilation. The outcome variable was ICU death.

Supplementary Table 4. Sensitivity analysis: cluster agreement under alternative transformations and clustering methods

Alternative specification	ARI vs. primary Ward solution	Cluster sizes
Ward linkage, log-transformed variables	0.660	177 / 47 / 3
Complete linkage	0.731	169 / 53 / 5
Average linkage	0.142	220 / 5 / 2
K-means (k=3)	0.407	139 / 77 / 11

Note: ARI = Adjusted Rand Index, comparing each alternative three-cluster solution to the primary solution (Ward linkage, squared Euclidean distance, untransformed standardized variables) reported in the main text. Log-transformation was applied to CRP, procalcitonin, D-dimer, and LDH prior to standardization, given their right-skewed distributions. All solutions were derived from the same nine standardized physiological variables (CRP, procalcitonin, D-dimer, INR, LDH, SpO2, PaO2, PaCO2, bicarbonate), N = 227.

Supplementary Table 5. Simplified adjusted model for ICU mortality, excluding invasive mechanical ventilation

Predictor	OR	95% CI	p value
Cluster 1 vs Cluster 2	3.578	1.661–7.710	0.001
Cluster 3 vs Cluster 2	7.900	0.402–155.207	0.174
Age (per year)	1.021	0.999–1.043	0.066
Sex	0.526	0.281–0.984	0.045
APACHE score	0.942	0.906–0.978	0.002
SOFA score	0.975	0.881–1.080	0.629

Note: Firth's penalized (bias-reduced) logistic regression, fit without invasive mechanical ventilation, which was the source of near-complete separation in the full model (Table 8). Reference category: Cluster 2. All confidence intervals are within a clinically interpretable range, unlike the full model.
